# *GALNT2* Expression Is Reduced in Patients with Type 2 Diabetes: Possible Role of Hyperglycemia

**DOI:** 10.1371/journal.pone.0070159

**Published:** 2013-07-22

**Authors:** Antonella Marucci, Lazzaro di Mauro, Claudia Menzaghi, Sabrina Prudente, Davide Mangiacotti, Grazia Fini, Giuseppe Lotti, Vincenzo Trischitta, Rosa Di Paola

**Affiliations:** 1 Research Unit of Diabetes and Endocrine Diseases, IRCCS “Casa Sollievo della Sofferenza”, San Giovanni Rotondo, Italy; 2 Blood Bank IRCCS “Casa Sollievo della Sofferenza”, San Giovanni Rotondo, Italy; 3 CSS-Mendel, Rome, Italy; 4 Department of Experimental Medicine, Sapienza University, Rome, Italy; University of Bremen, Germany

## Abstract

Impaired insulin action plays a major role in the pathogenesis of type 2 diabetes, a chronic metabolic disorder which imposes a tremendous burden to morbidity and mortality worldwide. Unraveling the molecular mechanisms underlying insulin resistance would improve setting up preventive and treatment strategies of type 2 diabetes. Down-regulation of GALNT2, an UDPN-acetyl-alpha-D-galactosamine polypeptideN-acetylgalactosaminyltransferase-2 (ppGalNAc-T2), causes impaired insulin signaling and action in cultured human liver cells. In addition, *GALNT2* mRNA levels are down-regulated in liver of spontaneously insulin resistant, diabetic Goto-Kakizaki rats. To investigate the role of GALNT2 in human hyperglycemia, we measured *GALNT2* mRNA expression levels in peripheral whole blood cells of 84 non-obese and 46 obese non-diabetic individuals as well as of 98 obese patients with type 2 diabetes. We also measured *GALNT2* mRNA expression in human U937 cells cultured under different glucose concentrations. *In vivo* studies indicated that *GALNT2* mRNA levels were significantly reduced from non obese control to obese non diabetic and to obese diabetic individuals (p<0.001). *In vitro* studies showed that *GALNT2* mRNA levels was reduced in U937 cells exposed to high glucose concentrations (i.e. 25 mmol/l glucose) as compared to cells exposed to low glucose concentration (i.e. 5.5 mmol/l glucose +19.5 mmol/l mannitol). In conclusion, our data indicate that *GALNT2* is down-regulated in patients with type 2 diabetes and suggest that this association is, at least partly, secondary to hyperglycemia. Further studies are needed to understand whether GALNT2 down-regulation plays a pathogenic role in maintaining and/or aggravating the metabolic abnormalities of diabetic milieu.

## Introduction

Type 2 diabetes is a chronic metabolic disorder imposing a tremendous burden to morbidity and mortality worldwide [1]. Impaired insulin action (i.e. insulin resistance, mainly in liver and skeletal muscle) plays a major role in the pathogenesis of type 2 diabetes [2], [3]. Thus, unraveling the molecular mechanisms underlying insulin resistance would improve setting up preventive and treatment strategies of type 2 diabetes. We have recently reported that GALNT2, an UDPN-acetyl-alpha-D-galactosamine polypeptideN-acetylgalactosaminyltransferase-2 (ppGalNAc-T2), modulates the expression of *ENPP1*, an inhibitor of insulin receptor signaling, thus becoming a new potential modulator of cellular insulin action [4]. In addition, *GALNT2* mRNA levels has been reported to be down-regulated in liver of spontaneously insulin resistant, diabetic Goto-Kakizaki rats as compared to control normoglycemic animals [5], thus strongly suggesting that GALNT2 has a role on insulin sensitivity and glucose homeostasis in rodents.

In order to get some insights about the role of GALNT2 expression in typical conditions of human insulin resistance and hyperglycemia, we measured *GALNT2* mRNA expression in peripheral whole blood cells (PWBC) of non-obese and non-diabetic individuals, obese non-diabetic insulin resistant individuals and patients with type 2 diabetes. We also investigated the effect of *in vitro* high glucose concentration on *GALNT2* expression in human cultured cells in order to address the biology underlying the expression changes we did observe in human studies.

## Results

### *GALNT2* mRNA Expression in Humans

*GALNT2* expression levels were measured in PWBC of 84 non-obese non-diabetic individuals, 46 obese non-diabetic individuals and 98 obese patients with type 2 diabetes. Salient clinical features of study subjects are shown in Table 1. Patients with type 2 diabetes were older than non-obese non-diabetic (p<0.001) as well as than obese non-diabetic (p<0.001) individuals. Diabetic patients were treated either with only diet (n = 5; 5.1%) or with oral hypoglycemic agents (OHA; n = 48; 49.0%) or with insulin ± OHA (n = 45; 45.9%). In addition, most of them were on anti-hypertensive (n = 69, 70.4%) and/or anti-dyslipidemic (n = 74; 75.5%) treatments. In contrast, no treatments at all were ongoing in non diabetic individuals.

**Table 1:** Clinical characteristics of study subjects.

	Non Obese subjects (n = 84)	Obese subjects (n = 46)	Diabetic patients (n = 98)
**Males (%)**	60 (71.4%)	32 (69.6%)	66 (67.3%)
**Age (yrs)**	41.4±11.3	47.1±11.3	55.9±9.6
**BMI (kg/m^2^)**	24.6±2.1	31.3±2.6	31.1±6.0
**Fasting Glucose (mg/dl)**	80.6±11.9	83.3±13.7	
**HOMA _IR_**	2.8±3.6	4.5±5.4	
**HbA_1C_ (%)**			8.0±1.7

Continuous variables were reported as mean ± SD whereas categorical variables as total frequency and percentages. BMI: body mass index; HbA1c: glycated hemoglobin.

**Figure 1:**
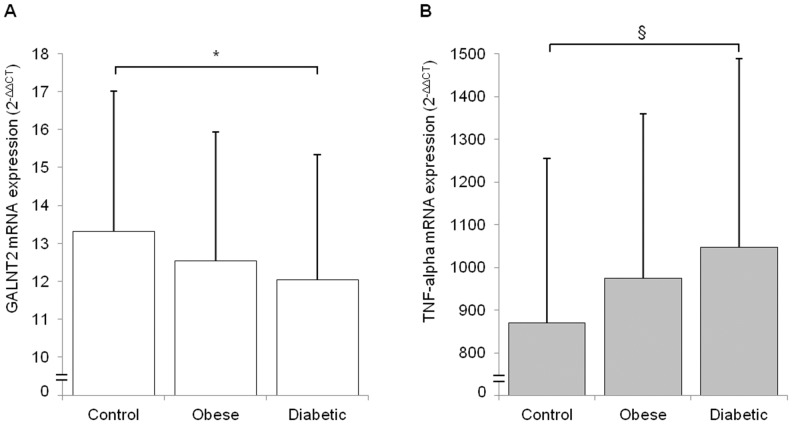
*GALNT2* and TNF-alpha expression in human PWBC. *GALNT2* (panel A) and *TNF-alpha* (panel B) mRNA expression levels were measured in PWBC of 84 non-obese non-diabetic individuals (Control), 46 obese non-diabetic individuals (Obese), and 98 obese patients with type 2 diabetes (Diabetic) as described in Methods. Bars represent mRNA levels expressed as 2^–ΔΔCT^. Data are means±SD. *p<0.05 ^§^p<0.005.

*GALNT2* mRNA levels, as normalized for *GAPDH* expression, were progressively reduced from non-obese non-diabetic individuals, to obese non-diabetic individuals and to obese patients with type 2 diabetes (p for trend <0.001). Such trend was still significant after taking into account age as a possible confounder, as well as ongoing treatments (p<0.001). Similar results were obtained when *GALNT2* expression was normalized for geometric mean of mRNA levels of *GAPDH*, *B-actin* and *18S* genes. Also in this case, a progressive reduction of *GALNT2* mRNA across the three study groups was observed (p for trend <0.01, Figure 1), with values in diabetic patients being significantly reduced as compared to that of those in non-obese control subjects (p<0.05). *TNF-alpha*, was then used as a positive control because of its reported over-expression in the obesity/hyperglycemic condition [6]. In fact, a progressive increase of *TNF-alpha* mRNA levels from controls, to obese non-diabetic subjects, to obese diabetic patients was observed (p for trend <0.005, Figure 1), with values in diabetic patients being significantly higher (p<0.005) as compared to those of non-obese control subjects. Of note, a significant positive correlation was observed between *TNF-alpha* and BMI in the whole sample of 228 individuals pooled together (r = 0.195, p = 0.003), thus resembling previous observations on the relationship between *TNF-alpha* and adiposity [7], [8], [9].

### Effect of High Glucose Concentration on *GALNT2* mRNA Expression Levels *in vitro*

**Figure 2:**
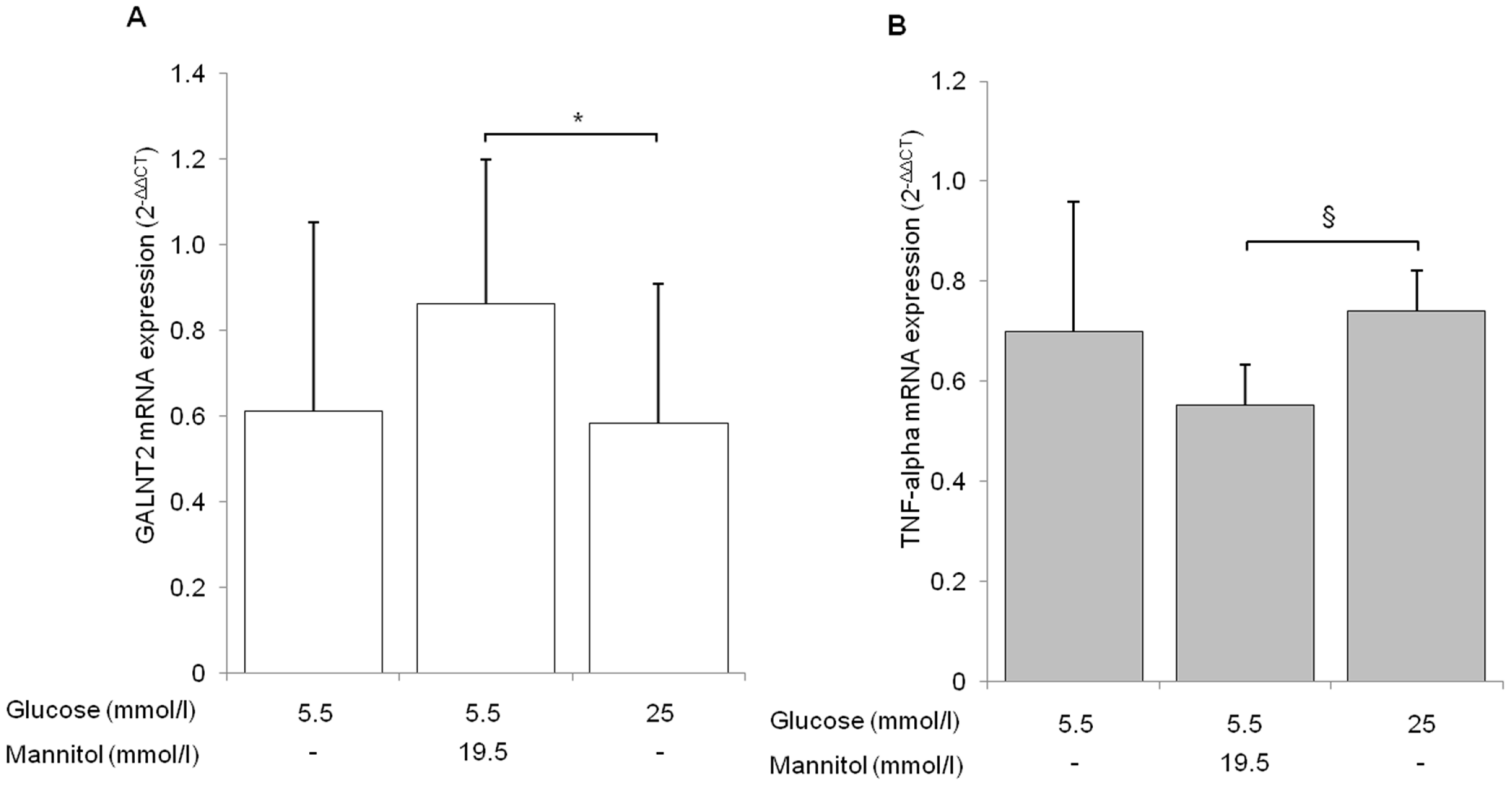
Glucose effect on *GALNT2* and TNF-alpha expression in U937 cells. U937 cells were treated for 24 h either with different glucose concentration (i.e. 5.5 mmol/l or 25 mmol/l), or with 5.5 mmo/l glucose plus 19.5 mmo/l mannitol. *GALNT2* (panel A) and *TNF-alpha* (panel B) mRNA expression levels were assesed as described in Methods. Bars represent quantitative analysis of mRNA levels related to low glucose treated cells of the first of six experiments (2^−ΔΔCT^). Data are means±SD of 6 experiments in separate times. *p<0.05 ^§^p<0.005.

To get deeper insights about the relationship between hyperglycemia and *GALNT2* down-regulation observed in human PWBC, we evaluated *in vitro* the effect of high glucose concentrations on *GALNT2* mRNA levels in cultured human U937 monocytes. Cells were pre-incubated for 24 h with increasing (i.e. 5.5, 15 and 25 mmol/l) glucose concentrations or with 5.5 mmo/l glucose plus either 9.5 mmol/l or 19.5 mmo/l mannitol, to control for increased osmolarity. When normalized for *GAPDH* expression, *GALNT2* mRNA levels in cells exposed to 25 mmol/l glucose were significantly reduced as compared to their appropriate control, namely cells exposed to 5.5 mmol/l glucose plus 19.5 mmol/l mannitol (35% reduction, p<0.05). Similar results were obtained when *GALNT2* expression was normalized for geometric mean of mRNA levels of *GAPDH*, *B-actin* and *18S* genes (Figure 2). In contrast, no difference was observed between cells incubated with 15 mmol/l and cells incubated with 5.5 mmol/l glucose plus 9.5 mmol/l mannitol (data not shown).

Levels of *TNF-alpha* mRNA, utilized as positive control, were increased in 25 mmol/l glucose treated cells as compared to cells treated with 5.5 mmol/l glucose plus 19.5 mmol/l mannitol (p<0.005, Figure 2).

No changes in GALNT2 protein expression levels were observed across the different experimental conditions. The apparent discordance between changes in mRNA and protein levels might be due to the short term (24 h)-treatment which could be not sufficient to cause variation in GALNT2 protein level.

## Discussion

The main finding of our study is that reduced *GALNT2* expression in circulating blood cells is associated with type 2 diabetes. Our present finding is perfectly in line with that reporting *GALNT2* down-regulation in liver of Goto-Kakizaki diabetic rats [5], thus pointing to hyperglycemia as a major cause of *GALNT2* down-regulation in patients with type 2 diabetes. This possibility is reinforced by our present finding that in human cultured monocytes, increasing glucose concentrations caused *GALNT2* down-regulation, as compared to appropriate control cells. Although caution has to be used in interpreting and extrapolating to the *in vivo* model data obtained in U937 cells, taken together, present and previous [5] data suggest a direct deleterious role of high glucose concentration on *GALNT2* expression. Since *GALNT2* down-regulation causes cellular insulin resistance [4], it could be hypothesized that it plays a central role on hyperglycemia-induced insulin resistance (i.e. glucose toxicity) [10].

As far as the mechanisms underlying the observed association is concerned, it is of note that GalNAc-T2, coded by *GALNT2*, is responsible for O-linked glycosylation, allowing the transfer of *N*-acetylgalactosamine from UDP-GalNAc to the hydroxyl group of a serine or threonine residue [11]. Such glycosylation has been reported to play an important role on insulin resistance and diabetes, either by competing for insulin-stimulated phosphorylation of effector molecules, or by directly regulating central components of insulin signaling, including IRS1 and Akt [12], [13], [14], [15], though the exact mechanism remains to be unraveled. In addition, very recent data from our laboratory indicate that *GALNT2* down-regulation (possibly by up-regulating ENPP1, a selective inhibitor of insulin-receptor function) reduces insulin signaling and action in cultured human liver cells [4]. Unfortunately, U937 cells are not responsive in terms of insulin signaling and action, thus making impossible to address such potential functional consequences of high glucose-induced *GALNT2* down-regulation also in these cells.

Further support to a role of *GALNT2* as a mediator of intermediate metabolism comes from studies showing that genetic variability at the *GALNT2* locus is associated with HDL-c and triglycerides levels [16], [17], [18], two main components of the insulin resistance/metabolic syndrome.

Our study has several strengths, including the relatively high number of study individuals when addressing gene expression in humans and the use of an *in vitro* model to get insights about the nature of the association we observed *in vivo*. In addition, our data were strengthened by the use of three different reference genes, as well as a positive control gene, namely *TNF-alpha*, which has been repeatedly reported to be up-regulated in patients with type 2 diabetes and *in vitro* high glucose conditions [6], [9].

We acknowledge that our study has also some limitations as follows. Although PWBC are an easily obtainable cell model which has been satisfactorily used as a good surrogate for studying gene expression changes in conditions of human insulin resistance and related abnormalities [19], [20], [21], [22], they do not represent a typical insulin target tissue. Thus, our present data have to be considered as a preliminary, hypothesis generating finding that needs to be deeper addressed by additional studies in typical insulin target tissues which are relevant for glucose homeostasis.

In conclusion, presently available *in vivo* data in both humans and rodents clearly indicate that *GALNT2* is down-regulated in insulin resistant diabetic individuals and suggest, together with *in vitro* data, that such an association is, at least partly, secondary to hyperglycemia. Further studies are needed to understand whether *GALNT2* down-regulation plays a pathogenic role in maintaining and/or aggravating the metabolic abnormalities of diabetic milieu.

## Methods

### Ethics Statement

The study and the informed consent procedures were approved by the local Institutional Ethic Committee IRCCS (Istituto di Ricovero e Cura a Carattere Scientifico) “Casa Sollievo della Sofferenza” and performed according to the Helsinki Declaration. All participants gave written consent.

### Subjects

Blood samples were obtained from 84 non-obese and non-diabetic individuals, 46 obese (i.e. BMI>28 Kg/m^2^) non-diabetic individuals, and 98 obese patients with type 2 diabetes who were recruited at the IRCCS “Casa Sollievo della Sofferenza” (S. Giovanni Rotondo, Gargano, Italy) as a part of an ongoing project on the genetics of type 2 diabetes and its chronic complications. The diagnosis of diabetes was based upon fasting glucose levels (i.e.>126 mg/dl) [23]. All diabetic subjects were studied after an overnight fast.

### Measurements

Plasma glucose was measured by the glucose oxidase method on a Beckman Glucose Analyzer 2 (Beckman Coulter, Inc., Fullerton, CA), serum insulin was measured by microparticle enzyme immunoassay (Abbott IMx Insulin Assay, Abbott Laboratories, Abbott Park, IL), and lipid profile (total serum cholesterol, HDL cholesterol and serum triglycerides) were measured by enzymatic method, Cobas, Roche Diagnostic, Welwin Garden City, Herts, UK.

The insulin resistance index homeostasis model assessment (HOMA_IR_) was calculated as fasting serum insulin (pmol/l)×fasting plasma glucose (mmol/l)/22.5.

### RNA Extraction, cDNA Synthesis, and Gene Expression Analysis

Total RNA from PWBC was isolated by using PAXgene Blood RNA Kit according to the manufacturer’s instruction (Qiagen S.r.l., Milano, Italy). Total RNA from U937 cells was isolated by using RNeasy Mini kit (Qiagen S.r.l., Milan, Italy). cDNA was generated by reverse transcription with iScript™ Reverse Transcription (Biorad, Hercules, CA) according to the manufacturer’s instructions and used as template in the subsequent analyses. Gene Expression Assay on Demand Kit Reagents (Applera Life Technologies, Carlsbad, CA) were used to quantify relative gene expression levels of *GALNT2*, *TNF-alpha, GAPDH* (glyceraldehyde 3-phosphate dehydrogenase), *B actin* and *18S* on ABI-PRISM 7500 (Applera Life Technologies, Carlsbad, CA). Expression levels of *GALNT2* were calculated by using the comparative ΔCT method. Briefly, for *GALNT2* expression in PWBC, the amount *GALNT2* was normalized both to *GAPDH* only or to *GAPDH, B actin* and *18S* considered together (geometric mean) [24] and related to a control RNA as calibrator (2^−ΔΔCT^). For *in vitro* experiments the amount of *GALNT2* was normalized as described above in experiments run in triplicate and related to control cells of the first of several experiments (2^−ΔΔCT^). Of note, across different experimental conditions (both *in vivo* and *in vitro*), values of i) expression stability of *GAPDH*, *B actin* and *18S* and ii) coefficient of variation of their normalized relative quantities (both assessed by Qbase+ software, Biogazelle, Belgium [24]) were <0.5 and <0.2, respectively, thus indicating no changes due to hyperglycemia/high glucose concentration conditions. This allows us using all three genes as reference target to normalize *GALNT2* and *TNF-alpha* expression levels across different experimental conditions.

### Cell Culture and Effect of High Glucose Concentration on *GALNT2* mRNA Expression Levels

U937 (human lymphoma cells) were routinely maintained in DMEM (5.5 mmo/l glucose) supplemented with 10% FBS, at 37°C and 5% CO_2_. After grown to 80% confluence, cells were starved for 24 h with DMEM supplemented with 0.5% FBS, and then incubated with DMEM 5.5 mmo/l, DMEM 15 mmo/l or DMEM 25 mmo/l glucose. To control for increased osmolarity due to incubation with glucose, cells were incubated with DMEM 5.5 mmo/l glucose plus either 9.5 mmol/l or 19.5 mmo/l mannitol for 24 h. After treatment with different glucose concentrations, *GALNT2* mRNA expression levels were measured and calculated as described above.

### Statistical Analyses

Differences between mean values were evaluated by unpaired or paired Student’s *t* test, as appropriate. Relationships between variables were evaluated by univariate or multivariate analysis, as appropriate. Data are presented as means±SD. SPSS 13 software package was used for all analyses.
